# Synthesis and ^11^C-Radiolabelling of 2-Carboranyl Benzothiazoles

**DOI:** 10.3390/molecules20057495

**Published:** 2015-04-23

**Authors:** Kiran B. Gona, Jaya Lakshmi V. N. P. Thota, Zuriñe Baz, Vanessa Gómez-Vallejo, Jordi Llop

**Affiliations:** 1 Radiochemistry and Nuclear Imaging Group, CIC biomaGUNE, Paseo Miramon 182, Parque Tecnológico de San Sebastián, San Sebastián 20009, Spain; E-Mails: kbgona@cicbiomagune.es (K.B.G.); vnpgona@cicbiomagune.es (J.L.V.N.P.T.); zbaz@cicbiomagune.es (Z.B.); 2 Radiochemistry Platform, CIC biomaGUNE, Paseo Miramón 182, Parque Tecnológico de San Sebastián, San Sebastián 20009, Spain; E-Mail: vgomez@cicbiomagune.es

**Keywords:** *m*-carborane, benzothiazole, positron emission tomography, radiolabelling, carbon-11

## Abstract

Dicarba-*closo*-dodecaboranes, commonly known as carboranes, possess unique physico-chemical properties and can be used as hydrophobic moieties during the design of new drugs or radiotracers. In this work, we report the synthesis of two analogues of 2-(4-aminophenyl)benzothiazole (a compound that was found to elicit pronounced inhibitory effects against certain breast cancer cell lines *in vitro*) in which the phenyl ring has been substituted by a *m*-carborane cage. Two different synthetic strategies have been used. For the preparation of 1-(9-amino-1,7-dicarba-*closo*-dodecaboran-1-yl)-benzo-thiazole, the benzothiazole group was first introduced on one of the cluster carbon atoms of *m*-carborane and the amine group was further attached in three steps. For the synthesis of 1-(9-amino-1,7-dicarba-*closo*-dodecaboran-1-yl)-6-hydroxybenzothiazole, iodination was performed before introducing the benzothiazole group, and the amino group was subsequently introduced in six steps. Both compounds were radiolabelled with carbon-11 using [^11^C]CH_3_OTf as the labelling agent. Radiolabelling yields and radiochemical purities achieved should enable subsequent *in vitro* and* in vivo* investigations.

## 1. Introduction

Outstanding advances in the early diagnosis and treatment of cancer have raised the 5-year relative survival rate for all cancers combined from 50% (1974) to 68% (2007) [1], however, cancer still accounted for 8.2 million deaths worldwide in 2012 [2]. Hence, the identification of novel structures useful for the design of new, potent, selective and less-toxic anticancer agents is still a major challenge to medicinal chemistry researchers.

Benzothiazoles are fused bicyclic systems and show interesting biomedical properties such as neuron protective [3,4], anti-malarial [5], and anti-inflammatory [6,7] effects, among others [8]. However, one of the most promising fields for benzothiazole derivatives is the development of anti-cancer drugs. Indeed, the benzothiazole moiety with various substitutions shows anti-tumour activity and a series of potent and selective anti-tumour agents have been developed to date [9]. Different mechanisms of action are involved in the anti-cancer properties of substituted benzothiazoles, as they can act as replication and mitosis inhibitors [10], topoisomerase II inhibitors [11], tyrosine kinase inhibitors [12], and cytochrome P450 inhibitors [13].

**Figure 1:**
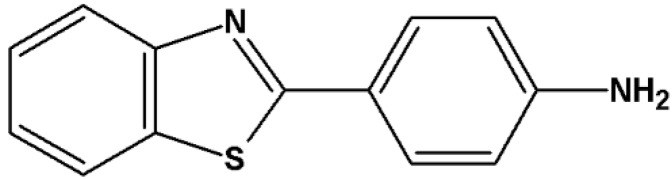
Chemical structure of CJM 126.

2-(4-Aminophenyl)benzothiazole (CJM 126, Figure 1), which was originally prepared as a synthetic intermediate for the preparation of polyhydroxylated 2-phenylbenzothiazoles, was found to elicit pronounced inhibitory effects against certain breast cancer cell lines *in vitro* [14]. Interestingly, structure-activity relationship studies performed with analogues of CJM 126 showed that substitution at the 3-position in the phenyl ring with an halogen atom or alkyl group enhanced potency in breast carcinoma and extended the *in vitro* spectrum to ovarian, lung, renal and colon carcinoma human cell lines [14]. However, replacement of halogen atoms with cyano- or hydroxy- substituents at the 3-position and introduction of chloro- substituent at the 2^1^-position of the aminophenyl group showed reduced activity compared to the parent amine CJM 126 [15]. These results suggest that small modifications in the structure severely influence the biological action of compounds in this series [16].

Dicarba-*closo*-dodecaboranes (commonly known as carboranes) are polyhedral clusters containing boron, hydrogen and carbon atoms, and have unique structural and chemical properties, e.g., rigid geometry, rich derivative chemistry, thermal and chemical stability and exceptional hydrophobic character. Because of this, carboranes have been used in different fields, including the preparation of heat-stable polymers [17], non-linear optics [18], and medicine [19]. In the context of medicinal applications, Endo and co-workers [20,21] showed that 1,2- and 1,7-dicarba-*closo*-dodecaborane (*o*- and *m*-carborane) can act as a hydrophobic structure of different biologically active molecules, because the carborane cage has a rotating volume similar to that of the phenyl group. Based on a similar approach, we recently reported the preparation of analogues of the D_2_ receptor antagonist raclopride, by replacing the poly-substituted phenyl moieties by different carborane clusters (*o*-carborane, *m*-carborane, and 1-methyl-*o*-carborane) [22], as well as the preparation of new analogues of rimonabant (a CB1 receptor antagonist first developed by Sanofi-Aventis [23] which has recently been approved in the European Union for the treatment of obesity) incorporating different carborane cages in their structure [24].

**Figure 2:**
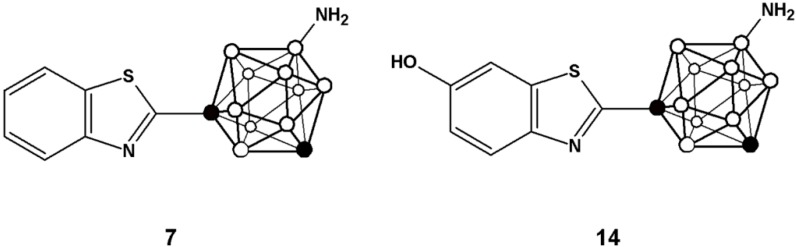
Chemical structure of the two analogues incorporating *m*-carborane developed in the current work.

Here, we report the preparation of two analogues of 2-(4-aminophenyl)benzothiazole in which the phenyl ring has been substituted by a *m*-carborane cage (compounds **7** and **14**, Figure 2). The inclusion of the carboranyl moieties may modulate the physic-chemical properties of the final compounds, which might result in an enhancement of the anti-tumour activity of the resulting benzothiazol derivatives. Moving towards* in vivo* applications, strategies for the preparation of the *N*-[^11^C]methylated derivatives of **7** and **14** have been implemented. Carbon-11 is a positron emitter with a half-life of 20.4 min; hence, the radiolabelled analogues might be used for the* in vivo* investigation in animal models using Positron Emission Tomography (PET), a molecular imaging technique with unparalleled sensitivity.

## 2. Results and Discussion

**Scheme 1:**
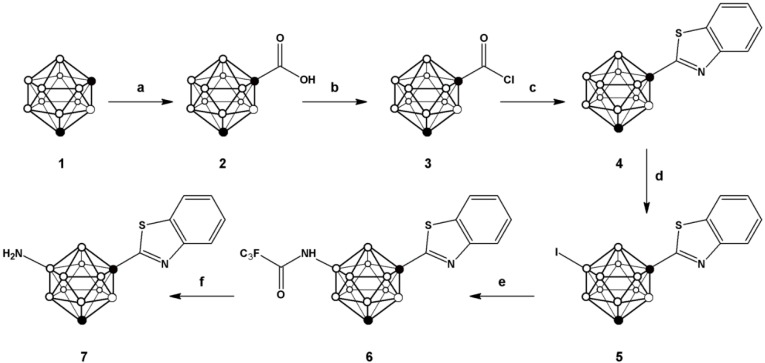
Schematic synthetic pathway followed for the preparation of compounds **2**–**7**; (**a**) *n*-BuLi, CO_2_; (**b**) PCl_5_; (**c**) 2-aminophenol, Lawesson’s reagent, toluene; (**d**) ICl, AlCl_3_; (**e**) trifluoroacetamide, K_3_PO_4_, tris(dibenzylideneacetone)dipalladium, Davephos, toluene; (**f**) NaOH.

One of the most convenient routes for the preparation of 2-substituted phenylbenzothiazoles consists of reacting benzoic acid with *o*-aminothiophenol in the presence of thionyl chloride [25], which leads to the* in situ* generation of the corresponding acid chloride and subsequent formation of the phenyl benzothiazole with excellent yields for a large collection of aromatic carboxylic acids. For the preparation of **7** we followed a similar strategy (see Scheme 1) but starting from *m-*carboranyl carboxylic acid (**2**), obtained by reaction of *m*-carborane (**1**) with n-BuLi and CO_2_. Further reaction with phosphorous pentachloride yielded the corresponding acid chloride **3**, which was refluxed with 2-aminothiophenol and 2,4-bis(4-methoxyphenyl)-1,3,2,4-dithiadiphosphetane-2,4-dithione (Lawesson’s reagent) in toluene to form 1-(1,7-dicarba-*closo*-dodecaboran-1-yl)-benzothiazole (**4**) in good overall yield (72.6%).

**Figure 3:**
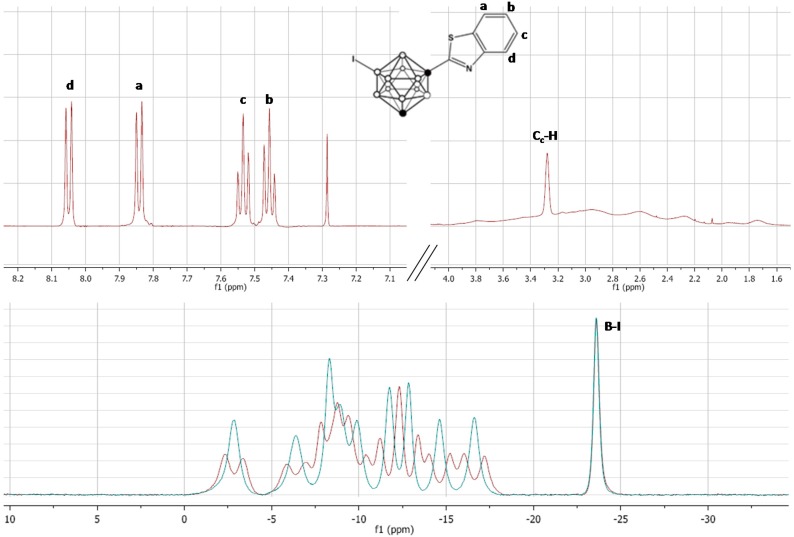
Top: ^1^H-NMR spectra for compound **5**; bottom: ^11^B- (**red**) and ^11^B{^1^H}-NMR (**blue**) spectra for compound **5**. The presence of four signals (integration 1:1:1:1) in the aromatic region and one signal (integration: 1) in the C_c_-H region (top) together with the presence of a singlet in the ^11^B-NMR spectra (−23.55 ppm) confirm the incorporation of the iodine atom on one of the boron atoms of the carborane cage. a, b, c and d identify the four protons in the aromatic ring and the corresponding signals in the ^1^H-NMR spectra.

In order to incorporate the amino group on the *m*-carborane cage, a previously reported methodology, based on the iodination with iodine monochloride (ICl) in the presence of aluminum chloride, was applied [26]. In principle, the incorporation of iodine can take place in different positions, either on the carborane cage or the aromatic ring. However, when one equivalent of iodine monochloride was used, the iodination took place preferentially on the *m*-carborane cage, leaving the phenyl ring un-substituted as confirmed by ^1^H-, and ^11^B- and ^11^B{^1^H}-NMR (Figure 3).

Boron atoms 9 and 10 have the highest electron density and hence these are the most susceptible positions to electrophilic halogenation [27]. In our case, the presence of only one boron atom which is a singlet both in ^11^B- and ^11^B{^1^H}-NMR spectra (integration pattern 1:1:1:1:1:1:1:1:1:1, see Figure 3) confirmed the mono-substitution on the carborane cage.

The trifluoroacetamide group was subsequently introduced by palladium-catalyzed Buchwald-Hartwig amidation reaction to yield compound **6**; subsequent hydrolysis under basic conditions using sodium hydroxide resulted in the formation of compound **7**, which was further used for the preparation of the N-[^11^C]methylated derivative (*vide infra*).

**Scheme 2:**
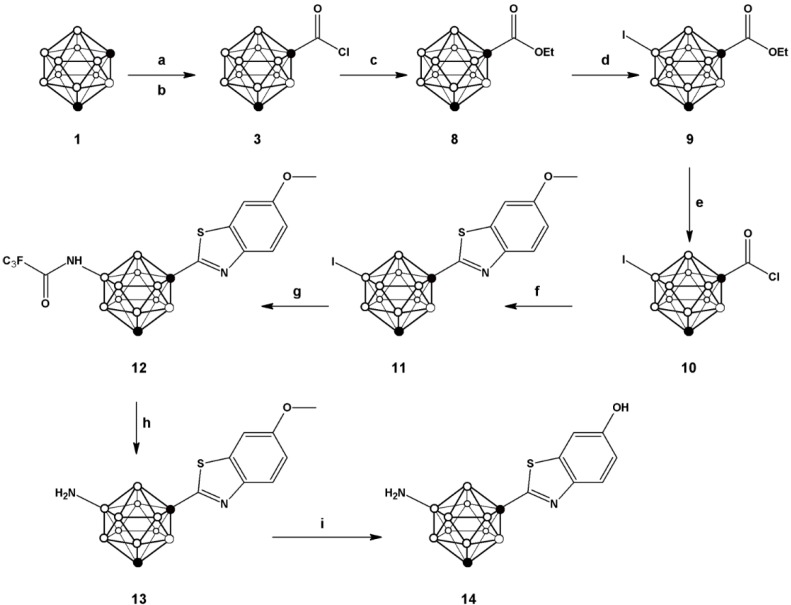
Schematic synthetic pathway followed for the preparation of compounds **8**–**14**; (**a**) *n*-BuLi, CO_2_; (**b**) PCl_5_; (**c**) ethanol; (**d**) ICl, AlCl_3_; (**e**) 2 N aqueous NaOH, PCl_5_; (**f**) 2-amino-5-methoxythiophenol, Lawesson’s reagent, toluene; (**g**) trifluoroacetamide, tris(dibenzylideneacetone)dipalladium, Davephos, K_3_PO_4_, toluene; (**h**) NaOH; (**i**) 1 M BBr_3_ in DCM.

The preparation of compound **14** (Figure 2) was first envisioned using a similar synthetic strategy. First, *m-*carboranyl carboxylic acid chloride (**3**) was prepared as above and further reacted with 2-amino-5-methoxythiophenol to yield 1-(1,7-dicarba-*closo*-dodecaboran-1-yl)-6-hydroxymethyl benzothiazole in good overall yield (71.2%). However, treatment of this compound with ICl resulted in preferential iodination on the phenyl ring, probably due to the electron-donnor character of the hydroxyl group which activates the *ortho* positions. Hence, an alternative synthetic route was approached; in this case, iodination was performed before attaching the substituted benzothiazole ring (Scheme 2). Briefly, the *m-*carboranyl carboxylic acid chloride (**3**) was esterified by treatment with ethanol, and subsequently iodinated using ICl in the presence of a catalytic amount of aluminum chloride to yield **9**.

**Scheme 3:**
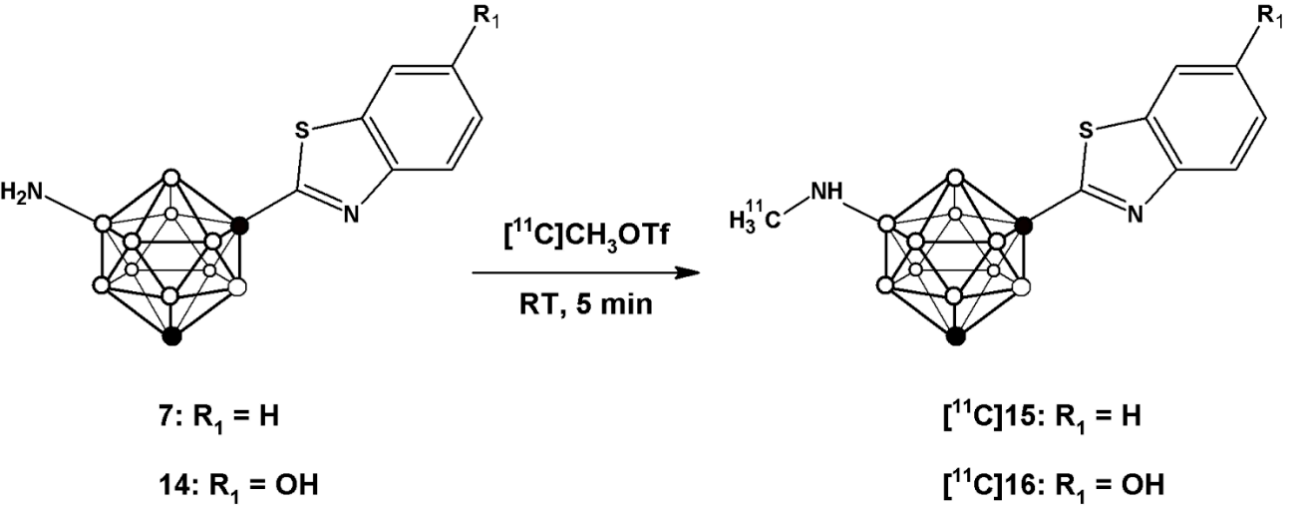
Schematic reaction for the preparation of [^11^C]**15** and [^11^C]**16** using [^11^C]CH_3_OTf as the labelling agent.

Compound **10**, obtained after hydrolysis and treatment with phosphorous pentachloride, was reacted with 2-amino-5-methoxythiophenol to yield **11**. Buchwald-Hartwig amidation using trifluoro acetamide followed by hydrolysis produced compound **13**, which after O-demethylation yielded compound **14** with overall yield of 6.4% (starting from compound **1**). This compound was also used for subsequent radiolabelling (*vide infra*). Radiolabelling of compounds **7** and **14** to yield [^11^C]**15** and [^11^C]**16** (Scheme 3) was achieved by treatment of the appropriate precursor with [^11^C]CH_3_OTf, which was synthesized with high specific activity following a well-established methodology in our laboratory [28]. The methylation reaction was conducted in a 2 mL stainless steel loop at room temperature, previously charged with a solution of the precursor (**7** or **14**).

Initially, the methylation reaction was attempted using [^11^C]CH_3_I as the labeling agent and DMSO as the solvent. Radiochemical conversion values below 30% (as calculated from chromatographic profiles) were obtained when 10M aqueous NaOH was used as the base, irrespective of the amount of base (2–20 µL) and the reaction time (1–10 min). In our configuration, in-loop reactions have to be conducted at room temperature, and hence [^11^C]CH3OTf, which is a better methylating agent, was considered as a suitable alternative. When this labeling agent was used, radiochemical conversion values of 72% ± 4% and 67% ± 7% were obtained for **7** and **14**, respectively, when the reaction was carried out for 5 min at room temperature using 1 mg of precursor; interestingly, the addition of a base was not required.

Complete syntheses for both molecules were conducted including purification (carried out by High Performance Liquid Chromatography—HPLC). The collected fractions (retention times of 9.2 and 6.4 min for [^11^C]**15** and [^11^C]**16**, respectively, see Supplementary Figures S1 and S3) were reformulated by trapping in a C-18 cartridge, further elution with ethanol and reconstitution with physiologic saline solution. Average production times from the end of the bombardment were 36 and 32 min for [^11^C]**15** and [^11^C]**16**, respectively; decay corrected radiochemical yields were 31.7% ± 5.6% and 20.5% ± 6.1%, respectively, resulting in an average final amount of radioactivity of 1.85 and 1.24 GBq for [^11^C]**15** and [^11^C]**16**, respectively, when the starting activity ([^11^C]CH4) was 18.5 GBq. The radiochemical purity at the end of the synthesis, as determined by HPLC (see Experimental Section, retention times of 11.5 and 9.0 min for [^11^C]**15** and [^11^C]**16**, respectively) was >98% (Supplementary Figures S2 and S4). Specific radioactivity values, according to historical values obtained in our laboratory with the same automatic configuration, were estimated to be in the range 80–120 GBq/μmol (EOS). Identification of the labelled species was carried out by HPLC-MS on the collected fractions after complete decay. The measured mass of the molecules were detected as [M+H]^+^ (*m/z* = 307.2 and retention time = 9.51 min for [^11^C]**15**; *m/z* = 323.2 and retention time = 8.39 min for [^11^C]**16**; Theoretical *m/z* = 307.4 and 323.4, respectively, Supplementary Figures S1 and S3). Yield, specific activity, and radiochemical purity values obtained at the end of the synthetic process should enable subsequent *in vivo* investigations in animal models.

## 3. Experimental Section

### 3.1. General

1,7-Dicarba-*closo*-dodecaborane was purchased from Katchem Ltd. (Prague, Czech Republic); All other reagents and anhydrous solvents, stored over 4 Å molecular sieves, were purchased from Aldrich Chemical Co. (Madrid, Spain) and used without further purification. HPLC grade solvents (ethanol, methanol, and acetonitrile) were purchased from Scharlab (Sentmenat, Barcelona, Spain) and used as received. Reactions were performed under inert atmosphere. Analytical thin layer chromatography (TLC) measurements were conducted with silica gel 60 F_254_ plates (Macherey-Nagel, Hoerdt, France). Visualization was accomplished with a UV source (254, 365 nm) and by treatment with an acidic solution of 1% PdCl_2_ in methanol. Carboranes charred as black spots on TLC. Manual chromatography was performed with silica gel 60 (70–230 mesh) from Scharlau (Sentmenat, Spain). ^1^H-, ^11^B-, ^11^B{^1^H}- and ^13^C-NMR (with complete proton decoupling) spectra were recorded on a 500-MHz Avance III Bruker spectrometer. Chemical shifts were reported in ppm with the solvent resonances as the internal standard (CHCl_3_: δ 7.26 ppm for ^1^H and δ 77.0 ppm for ^13^C). UPLC/ESI-MS analyses were performed using an AQUITY UPLC separation module coupled to a LCT TOF Premier XE mass spectrometer (Waters, Manchester, UK). An Acquity BEH C_18_ column (1.7 µm, 5 mm, 2.1 mm) was used as stationary phase. The elution buffers were A (water and 0.1% formic acid) and B (Methanol and 0.1% formic acid). The detection was carried out in both positive & negative ion modes, monitoring the most abundant isotope peaks from the mass spectra.

### 3.2. Synthesis of 1,7-Dicarba-closo-dodecarborane-1-carboxylic acid (**2**) and 1,7-Dicarba-closo-dodecarborane-1-carboxylic acid chloride (**3**)

Compounds **2** and **3** were prepared following previously reported methods [29]. Briefly, m-carborane (3.0 g, 20.97 mmol) was dissolved in diethyl ether (150 mL) and treated with n-BuLi (14.4 mL, 23.07 mmol, 1.6 M in hexane) at −78 °C. The resulting mixture was stirred for 20 min and then dry ice (7.5 g) was added. The mixture was brought to room temperature and further stirred for 1 h. The solvent was removed under reduced pressure, water (100 mL) was added to the residue and unreacted m-carborane was extracted with hexane (2 × 50 mL). The aqueous phase was acidified with 3 N HCl and extracted with hexane (4 × 40 mL). The organic layers were combined, dried over Na_2_SO_4_, and concentrated to yield 1,7-dicarba-*closo*-dodecarborane-1-carboxylic acid (**2**, 3.52 g, 90% yield) as a white solid. ^1^H-NMR (500 MHz, CDCl_3_): δ 8.89, (1H, bs, COOH), 3.05 (1H, s, cage C-H), 3.70–1.20 (10H, m, B_10_H_10_); ^13^C-NMR (126 MHz, CDCl_3_): 167.15, 71.16, 54.85; ^11^B-NMR (160 MHz, CDCl_3_): −4.87 (d, 1B), −6.49 (d, 1B), −10.52 (d, 2B), −11.25 (d, 2B), −13.15 (d, 2B), −15.63 (d, 2B). 1,7-Dicarba-*closo*-dodecarborane-1-carboxylic acid (3.0 g, 16.04 mmol) was dissolved in toluene and reacted with phosphorous pentachloride (3.5 g, 16.84 mmol) at room temperature for 6 h. The reaction mixture was fractional distilled (120 °C, 5 mm Hg vacuum) to get 1,7-dicarba-*closo*-dodecarborane-1-carboxylic acid chloride (**3**, 2.46 g, 75% yield).

### 3.3. Synthesis of 1-(1,7-Dicarba-closo-dodecaboran-1-yl)-benzothiazole (**4**)

To a solution of 1,7-dicarba-*closo*-dodecarborane-1-carboxylic acid chloride (0.55 g, 2.67 mmol) in dry toluene (15 mL), 2-aminothiophenol (860 μL, 8.02 mmol) and Lawesson’s reagent (378 mg, 0.96 mmol) were added and the resulting mixture was refluxed for 14 h. The mixture was concentrated, water (100 mL) was added and the mixture was extracted with dichloromethane (3 × 50 mL). The combined organic layers were dried over Na_2_SO_4_, concentrated and purified using silica gel column chromatography (starting mobile phase: hexane; final mobile phase: 3% ethyl acetate) to afford 1-(1,7-dicarba-*closo*-dodecaboran-1-yl)-benzothiazole (**4**, 610 mg, 83% yield) as a yellow solid. ^1^H-NMR (500 MHz, CDCl_3_): 8.05 (1 H, dt, CH, phenyl), 7.83 (1 H, dt, CH, phenyl), 7.52 (1 H, ddd, CH, phenyl), 7.44 (1 H, ddd, CH, phenyl), 3.17 (1 H, d, cage C-H), 3.70–1.70 (10H, m, B_10_H_10_); ^13^C-NMR (126 MHz, CDCl_3_): 162.39, 152.57, 135.86, 126.73, 126.17, 123.83, 121.32, 73.37, 55.34; ^11^B-NMR (160 MHz, CDCl_3_): −3.82 (d, 1B), −7.51 (d, 1B), −10.26 (dd, 4B), −13.09 (d, 2B), −14.72 (d, 2B).

### 3.4. Synthesis of 1-(9-Iodo-1,7-dicarba-closo-dodecaboran-1-yl)-benzothiazole (**5**)

To a solution of 1-(1,7-dicarba-*closo*-dodecaboran-1-yl)-benzothiazole (0.50 g, 1.811 mmol) in dry dichloromethane (15 mL), aluminum chloride (50 mg, 0.37 mmol) and iodine monochloride (95 μL, 1.811 mmol) were added. The mixture was refluxed for 14 h, cooled to room temperature, and excess iodide was quenched with 5% sodium thiosulfate solution (50 mL). The products were extracted with dichloromethane (3 × 50 mL), the organic layers were combined, dried over Na_2_SO_4_, concentrated with a rotary evaporator and the crude was purified using silica gel column chromatography (starting mobile phase: hexane; final mobile phase: 3% ethyl acetate) to yield the title compound **5** (550 mg, 75% yield) as a yellow solid. ^1^H-NMR (500 MHz, CDCl_3_): 8.05 (1 H, dt, CH, phenyl), 7.84 (1 H, dt, CH, phenyl), 7.54 (1 H, ddd, CH, phenyl), 7.46 (1 H, ddd, CH, phenyl), 3.28 (1 H, s, cage C-H ), 3.70–1.70 (10H, m, B_10_H_10_); ^13^C-NMR (126 MHz, CDCl_3_): 161.02, 152.49, 135.85, 126.98, 126.47, 123.93, 121.45, 74.57, 56.27; ^11^B-NMR (160 MHz, CDCl_3_): −2.85 (d, 1B), −6.43 (d, 1B), −8.31 (d, 1B), −8.90 (d, 1B), −9.88 (d, 1B), −11.75 (d, 1B), −12.84 (d, 1B), −14.61 (d, 1B), −16.61 (d, 1B), −23.64 (s, 1B).

### 3.5. Synthesis of 1-(9-Trifluoroacetylamino-1,7-dicarba-closo-dodecaboran-1-yl)-benzothiazole (**6**)

To a solution of 1-(9-iodo-1,7-dicarba-*closo*-dodecaboran-1-yl)-benzothiazole (800 mg, 1.985 mmol), trifluoroacetamide (673 mg, 5.955 mmol) and tripotassium phosphate (2.1 g, 9.925 mmol) in dry toluene, tris(dibenzylideneacetone)dipalladium (45.5 mg, 0.049 mmol) and 2-dicyclohexyl-phosphino-2'-(*N*,*N*-dimethylamino)biphenyl (Davephos, 39 mg, 0.099 mmol) were added. The mixture was refluxed for 14 h, filtered, concentrated using rotary evaporator and the crude was purified using silica gel column chromatography (starting mobile phase: hexane; final mobile phase: 10% ethyl acetate) to afford 1-(9-trifluoroacetylamino-1,7-dicarba-*closo*-dodecaboran-1-yl)-benzothiazole (**6**, 630 mg, 81% yield) as a yellow solid. ^1^H-NMR (500 MHz, CDCl_3_): 8.05 (1 H, dt, CH, phenyl), 7.84 (1 H, dt, CH, phenyl), 7.53 (1 H, ddd, CH, phenyl), 7.46 (1 H, ddd, CH, phenyl), 5.93 (1 H, s, NH), 3.18 (1 H, s, cage C-H), 3.70–1.70 (10H, m, B_10_H_10_); ^13^C-NMR (126 MHz, CDCl_3_): 161.16, 152.49, 135.85, 126.95, 126.45, 123.92, 121.44, 70.80, 52.56; ^11^B-NMR (160 MHz, CDCl_3_): −1.71 (s, 1B), −4.30 (d, 1B), −7.91 (d, 1B), −10.88 (dd, 2B), −11.65 (d, 1B), −13.69 (d, 1B), −14.82 (d, 1B), −16.21 (d, 1B), −18.71 (d, 1B).

### 3.6. Synthesis of 1-(9-Amino-1,7-dicarba-closo-dodecaboran-1-yl)-benzothiazole (**7**)

To a solution of 1-(9-trifluoroacetylamino-1,7-dicarba-*closo*-dodecaboran-1-yl)-benzothiazole (150 mg, 0.38 mmol) in methanol (1.5 mL), tetrahydrofuran (1.5 mL), isopropanol (1.5 mL), water (6 mL) and sodium hydroxide (900 mg, 22.5 mmol) were added. The resulting mixture was stirred at room temperature for 72 h and concentrated below 30 °C. Extraction was carried out with ethyl acetate (3 × 50 mL), the organic layers were combined, dried over sodium sulfate, concentrated using rotary evaporator and the crude was purified using silica gel column chromatography (starting mobile phase: hexane; final mobile phase: 50% ethyl acetate) to afford 1-(9-amino-1,7-dicarba-*closo*-dodecaboran-1-yl)-benzothiazole (**7**, 35 mg, 31% yield) as a white solid. ^1^H-NMR (500 MHz, CDCl_3_): 8.03 (1 H, dt, CH, phenyl), 7.80 (1 H, dt, CH, phenyl), 7.50 (1 H, ddd, CH, phenyl), 7.42 (1 H, ddd, CH, phenyl), 5.19 (2H, bs, NH_2_), 3.03 (1 H, s, cage C-H ), 3.70–1.70 (10H, m, B_10_H_10_); ^13^C-NMR (126 MHz, CDCl_3_): 162.20, 152.52, 135.83, 126.75, 126.19, 123.81, 121.35, 69.40, 51.37; ^11^B-NMR (160 MHz, CDCl_3_): −1.72 (s, 1B), −4.04 (d, 1B), −8.05(d, 1B), −10.72 (dd, 2B), −13.89 (d, 1B), −15.24 (d, 1B), −16.90 (d, 1B), −20.50 (d, 1B), −22.92 (d, 1B); LCMS (ESI) Experimental [M+H]^+^*m/z* = 293.12 (theoretical value: 293.41).

### 3.7. Synthesis of 1,7-Dicarba-closo-dodecarborane-1-ethylcarboxylate (**8**)

A solution of 1,7-dicarba-*closo*-dodecarborane-1-carboxylic acid chloride (5.5 g, 26.7 mmol) was refluxed in ethanol (150 mL) for 4 h. After cooling, the mixture was concentrated to yield 1,7-dicarba-*closo*-dodecarborane-1-ethyl carboxylate (5.7 g, 98% yield) as brown viscous liquid, which was used without further purification. ^1^H-NMR (500 MHz, CDCl_3_): 4.20 (2 H, q, CH_2_CH_3_), 3.02 (1 H, s, cage C-H), 1.29 (3 H, t, CH_3_CH_2_), 3.70–1.70 (10 H, m, B_10_H_10_); ^13^C-NMR (126 MHz, CDCl_3_): 161.75, 63.75, 54.67, 13.76; ^11^B-NMR (160 MHz, CDCl_3_): −4.89 (d, 1B), −6.98 (d, 1B), −10.69 (d, 2B), −11.28 (d, 2B), −13.32 (d, 2B), −15.65 (d, 2B).

### 3.8. Synthesis of (9-Iodo-1,7-dicarba-closo-dodecarborane-1-yl)-ethyl carboxylate (**9**)

Prepared according to the procedure described for compound **5**; yield after purification: 78%. ^1^H-NMR (500 MHz, CDCl_3_): 4.22 (2H, q, CH_2_CH_3_), 3.15 (1H, s, cage C-H), 3.70–1.70 (10H, m, B_10_H_10_), 1.30 (3H, t, CH_3_CH_2_); ^13^C-NMR (126 MHz, CDCl_3_): 160.72, 73.65, 64.19, 55.64, 13.77. ^11^B-NMR (160 MHz, CDCl_3_): −3.84 (d, 1B), −5.91 (d, 1B), −8.55 (d, 1B), −10.12 (d, 1B), −11.15 (d, 1B), −11.99 (d, 1B), −13.12 (d, 1B), −15.59 (d, 1B), −17.58 (d, 1B), −23.76 (s, 1B).

### 3.9. Synthesis of (9-Iodo-1,7-dicarba-closo-dodecarborane-1-yl)-carboxylic acid chloride (**10**)

To a solution of (9-iodo-1,7-dicarba-*closo*-dodecarborane-1-yl)-ethylcarboxylate (4.3 g, 12.6 mmol) in ethanol (30 mL), 2 N NaOH solution (30 mL) was added and the mixture was stirred at room temperature for 30 min. The solvent was evaporated below 30 °C, and the residue was neutralized with 2 N HCl, extracted with ethylacetate (3 × 50 mL), and the combined organic layers were dried over sodium sulfate. After solvent evaporation, (9-iodo-1,7-dicarba-*closo*-dodecarborane-1-yl)-carboxylic acid (**18**, 3.99 g, 98% yield) was obtained. ^1^H-NMR (500 MHz, CDCl_3_): 9.35 (1H, bs, COOH), 3.18 (1H, s, cage C-H), 3.70–1.70 (10H, m, B_10_H_10_); ^13^C-NMR (126 MHz, CDCl_3_): 166.03, 72.25, 55.83; ^11^B-NMR (160 MHz, CDCl_3_): −3.81 (d, 1B), −5.82 (d, 1B), −8.50 (d, 1B), −10.09 (d, 1B), −11.12 (d, 1B), −11.93 (d, 1B), −13.05 (d, 1B), −15.56 (d, 1B), −17.54 (d, 1B), −23.74 (s, 1B). The acid chloride was prepared by reacting compound **18** (3.99 g, 12.7 mmol) dissolved in toluene (40 mL) with phosphorous pentachloride (2.88 g, 13.86 mmol) at room temperature for 6 h. The reaction mixture was concentrated under high vaccum and used for the next step without purification.

### 3.10. Synthesis of 1-(9-Iodo-1,7-dicarba-closo-dodecaboran-1-yl)-6-hydroxymethyl benzothiazole (**11**)

Compound **11** was prepared according to the procedure described for compound **4**, but 2-amino-5-methoxyphenol was used instead of 2-aminophenol, with a yield of 73.8% starting from the acid. ^1^H-NMR (500 MHz, CDCl_3_): 7.91 (1 H, d, CH, phenyl), 7.25 (1 H, d, CH, phenyl), 7.12 (1 H, dd, CH, phenyl), 3.89 (3 H, s, CH_3_O-), 3.26 (1 H, s, cage C-H), 3.70–1.70 (10 H, m, B_10_H_10_); ^13^C-NMR (126 MHz, CDCl_3_): 158.60, 158.12, 147.00, 137.34, 124.46, 116.59, 103.52, 74.71, 56.18, 55.87; ^11^B-NMR (160 MHz, CDCl_3_): −2.90 (d, 1B), −6.62 (d, 1B), −8.42 (d, 1B), −8.81 (d, 1B), −9.90 (d, 1B), −11.85 (d, 1B), −12.94 (d, 1B), −14.66 (d, 1B), −16.64 (d, 1B), −23.63 (s, 1B).

### 3.11. Synthesis of 1-(9-Trifluoroacetylamino-1,7-dicarba-closo-dodecaboran-1-yl)-6-hydroxymethyl benzothiazole (**12**)

Compound **12** was prepared according to the procedure described for compound **6**; yield 72%. ^1^H-NMR (500 MHz, CDCl_3_): 7.91 (1 H, d, CH, phenyl), 7.25 (1 H, d, CH, phenyl), 7.12 (1 H, dd, CH, phenyl), 5.93 (1 H, s, NH), 3.89 (3 H, s, CH_3_O-), 3.17 (1 H, s, cage C-H), 3.70–1.70 (10 H, m, B_10_H_10_); ^13^C-NMR (126 MHz, CDCl_3_): 159.49 (q), 158.60, 158.24, 146.99, 137.34, 124.44, 116.59, 103.50, 70.95, 55.85, 52.47; ^11^B-NMR (160 MHz, CDCl_3_): −1.77 (s, 1B), −4.33 (d, 1B), −8.09 (d, 1B), −10.93 (dd, 2B), −11.70 (d, 1B), −13.74 (d, 1B), −14.87 (d, 1B), −16.23 (d, 1B), −18.71 (d, 1B).

### 3.12. Synthesis of 1-(9-Amino-1,7-dicarba-closo-dodecaboran-1-yl)-6-hydroxymethyl benzothiazole (**13**)

Compound **13** was prepared according to the procedure described for compound **7**; yield 32%. ^1^H-NMR (500 MHz, CDCl_3_): 7.89 (1 H, d, CH, phenyl), 7.23 (1 H, d, CH, phenyl), 7.09 (1 H, dd, CH, phenyl), 3.87 (3 H, s, CH_3_O-), 3.46 (2 H, bs, NH_2_), 3.03 (1 H, s, cage C-H), 3.70–1.70 (10 H, m, B_10_H_10_); ^13^C-NMR (126 MHz, CDCl_3_): 159.25, 158.44, 146.98, 137.26, 124.32, 116.37, 103.51, 69.79, 55.83, 51.56; ^11^B-NMR (160 MHz, CDCl_3_): 3.77 (s, 1B), −4.53 (d, 1B), −8.38 (d, 1B), −10.57 (dd, 2B), 12.42 (d, 1B), −13.79 (d, 1B), −15.38 (d, 1B), −16.96 (d, 1B), −21.91 (d, 1B).

### 3.13. Synthesis of 1-(9-Amino-1,7-Dicarba-closo-dodecaboran-1-yl)-6-hydroxy benzothiazole (**14**)

A solution of 1-(9-amino-1,7-dicarba-*closo*-dodecaboran-1-yl)-6-hydroxymethyl benzothiazole (**13**, 30 mg, 0.088 mmol) in dry dichloromethane (1 mL) was cooled to 0 °C and 1 M solution of BBr_3_ in dichloromethane (0.44 mL, 0.44 mmol) was added dropwise. The mixture was allowed to stir at room temperature for 12 h. Sodium bicarbonate (10% aqueous solution, 1 mL) was added. The solution was extracted with dichloromethane (3 × 5 mL), the organic layers were combined, dried over sodium sulfate, and concentrated to yield 1-(9-amino-1,7-dicarba-closo-dodecaboran-1-yl)-6-hydroxy benzothiazole (**14**, 20 mg, 71% yield) as a white solid. ^1^H-NMR (500 MHz, CD_3_OD): 7.78(1 H, d, CH, phenyl), 7.25 (1 H, d, CH, phenyl), 7.03 (1 H, dd, CH, phenyl), 3.85 (1 H, bs, cage C-H), 3.83–1.70 (10 H, m, B_10_H_10_);^ 13^C-NMR (126 MHz, CD_3_OD): 154.48, 153.67, 143.41, 135.06, 122.41, 115.69, 105.39, 72.59, 55.92; ^11^B-NMR (160 MHz, CD_3_OD): 1.48 (s, 1B), −4.71 (d, 1B), −8.28 (d, 1B), −10.86 (dd, 2B), −12.01 (d, 1B), −13.67 (d, 1B), −14.88 (d, 1B), −16.35 (d, 1B), −20.71 (d, 1B);LCMS (ESI) Experimental [M + H]^+^*m/z* = 308.5 (theoretical value: 308.4).

### 3.14. Radiochemistry

[^11^C]CH_3_OTf synthesis was carried out using a TRACERlab FX_C_ Pro synthesis module (GE Healthcare, Milwaukee, WI, USA) following a well established procedure in our lab [28]. The [^11^C]CH_3_OTf was introduced in the reaction loop (AutoLoop^TM^ system, Bioscan Inc., Washington, DC, USA), pre-charged with a solution of the precursor and the solvent (total volume: 100 µL). The reaction was allowed to occur at room temperature. During process optimization, the crude reaction mixtures were directly collected in vials by pushing with acetonitrile and the resulting solutions were analyzed by radio-HPLC to obtain radiochemical conversion. For final runs under optimal conditions, the reaction mixture was directly pushed to a semi-preparative HPLC column (Meditarian Sea18 C18 column, 250 × 10 mm, 5 μm, Teknokroma, Sant Cugat del Vallés, Spain) using aqueous 0.1 M AMF (pH adjusted to 3.9 using HCOOH)/MeCN in a ratio of 50/50 as the mobile phase (flow rate of 3 mL/min). The desired fraction (retention time = 9.2 min & 6.4 min for [^11^C]**15** and [^11^C]**16** respectively on radiometric and UV detection) was collected and reformulated using solid phase extraction. The amount of radioactivity obtained was measured in a dose calibrator (PETDOSE HC, Comecer, Castel Bolognese, Italy). Radiochemical purity of the final compound was determined by radio-HPLC, using an Agilent 1200 Series HPLC system (Las Rozas, Madrid, Spain) equipped with a multiple wavelength UV detector (λ = 254 nm) and a radiometric detector (Gabi, Raytest, Straubenhardt, Germany). An Eclipse C18 column (4.6 × 150 mm, 5 μm particle size) was used as stationary phase and aqueous 0.1 M AMF (pH adjusted to 3.9 using HCOOH)/MeOH 55/45 as the mobile phase. The retention times of [^11^C]**15** and [^11^C]**16** were 11.5 and 9.0 min respectively. Identification of the desired species was carried out by HPLC-MS performed on the purified fraction after complete decay, using an AQUITY UPLC separation module coupled to a LCT TOF Premier XE mass spectrometer (Waters, Manchester, UK). An Acquity BEH C18 column (1.7 μm, 5 mm, 2.1 mm) was used as stationary phase. The elution buffers were MEOH (A) and 0.1% formic acid aqueous solution (B). The column was eluted with a gradient: *t* = 0min, 95% B; *t* = 0.5min, 95% B; *t* = 16 min, 1% B; and *t* = 20 min, 1% B. Total run length was 20 min; injection volume was 5 μL, and the flow rate was 0.3 mL/min. The detection was carried out in positive ion mode, monitoring the most abundant isotope peaks from the mass spectra. Compounds [^11^C]**15** and [^11^C]**16** were detected as a protonated species, *m/z* = 307.2 and *m/z* = 323.2 respectively.

## 4. Conclusions

The synthesis of 1-(9-amino-1,7-dicarba-*closo*-dodecaboran-1-yl)-benzothiazole could be achieved by reaction of *m-*carboranyl carboxylic acid chloride with 2-aminothiophenol and Lawesson’s reagent, followed by iodination using iodine monochloride in the presence of aluminum chloride, subsequent palladium-catalyzed Buchwald-Hartwig amidation reaction to incorporate the trifluoroacetamide group and final hydrolysis under basic conditions. The preparation of 1-(9-amino-1,7-dicarba-*closo*-dodecaboran-1-yl)-6-hydroxy benzothiazole required a different approach, based on the preparation of the iodinated *m-*carboranyl carboxylic acid chloride in a first step, followed by reaction with 2-amino-5-methoxythiophenol, Buchwald-Hartwig amidation, hydrolysis and finally *O*-demethylation. Both compounds could be efficiently labeled with carbon-11 to yield the corresponding *N*-[^11^C]methylated derivatives, which might be used for subsequent* in vivo* investigation using Positron Emission Tomography. Future work will be devoted to: (i) create a library of analogues of compounds **7** and **14**; (ii) determine the inhibitory effects of the novel compounds against cancer cell lines *in vitro*; (iii) perform toxicological studies of the new compounds; and (iv) proceed to* in vivo* investigation of the most promising compounds in animal models.

## Supplementary Materials

Supplementary materials can be accessed at: http://www.mdpi.com/1420-3049/20/05/7495/s1.
