# Supplementary material for: Synthesis and 11C-Radiolabelling of 2-Carboranyl Benzothiazoles
Source: Molecules. 2015 Apr 23;20(5):7495–508. doi: 10.3390/molecules20057495 (PMC6272141; doi:10.3390/molecules20057495)
Supplement: Supplementary file 1 [file molecules-20-07495-s001.pdf]

Supplementary Materials

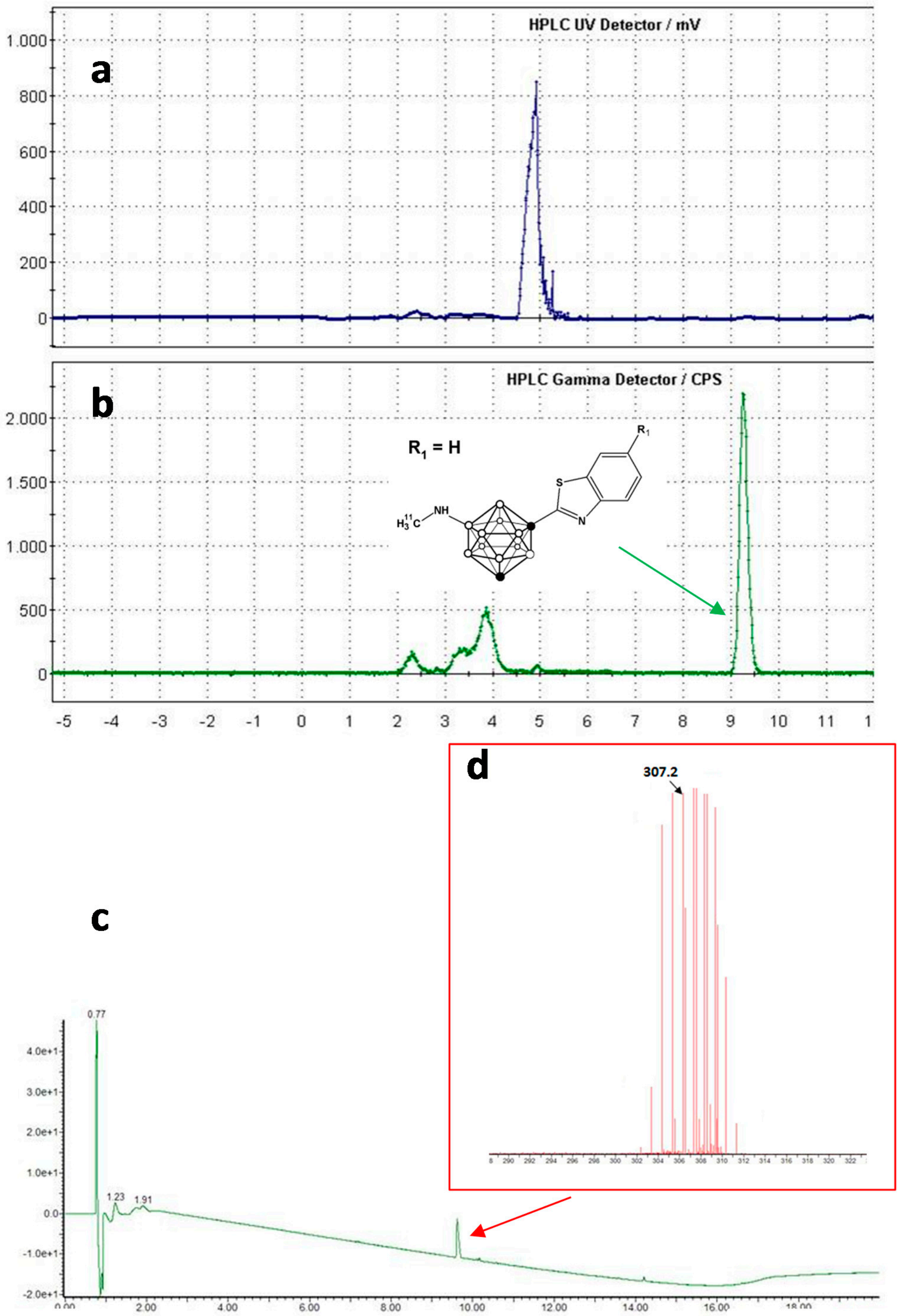

Figure S1. (**a**,**b**) Chromatographic profiles corresponding to the purification of [^{11}C]**15**: (a) UV detector and (b) radiometric detector. The collected fraction is indicated with an arrow in the radiometric profile; (**c**) chromatographic profile corresponding to LC-MS analysis of pure [^{11}C]**15** after complete decay; (**d**) MS spectrum for maximum intensity peak.

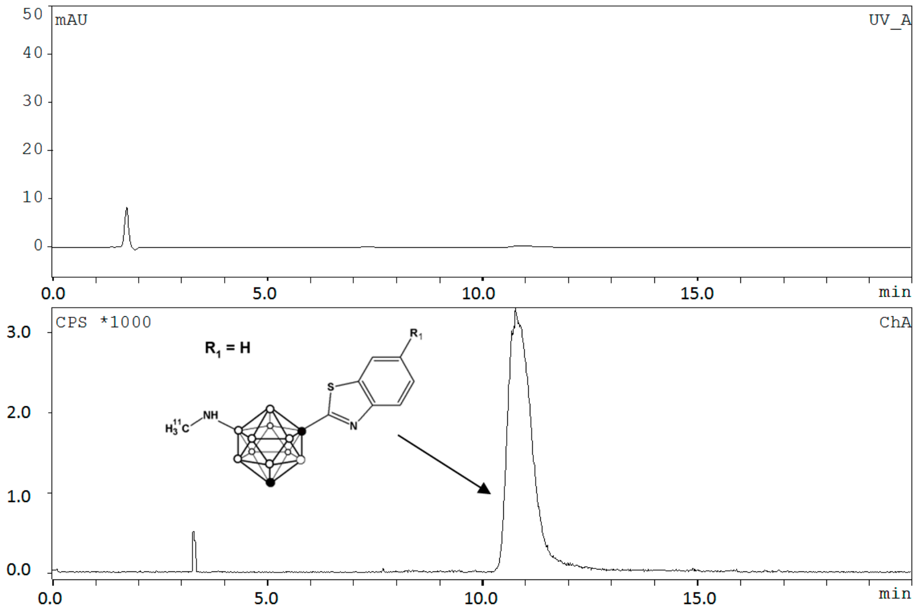

Figure S2. Chromatographic profiles corresponding to the quality control of [^{11}C]**15**: UV detector (top) and radiometric detector (bottom).

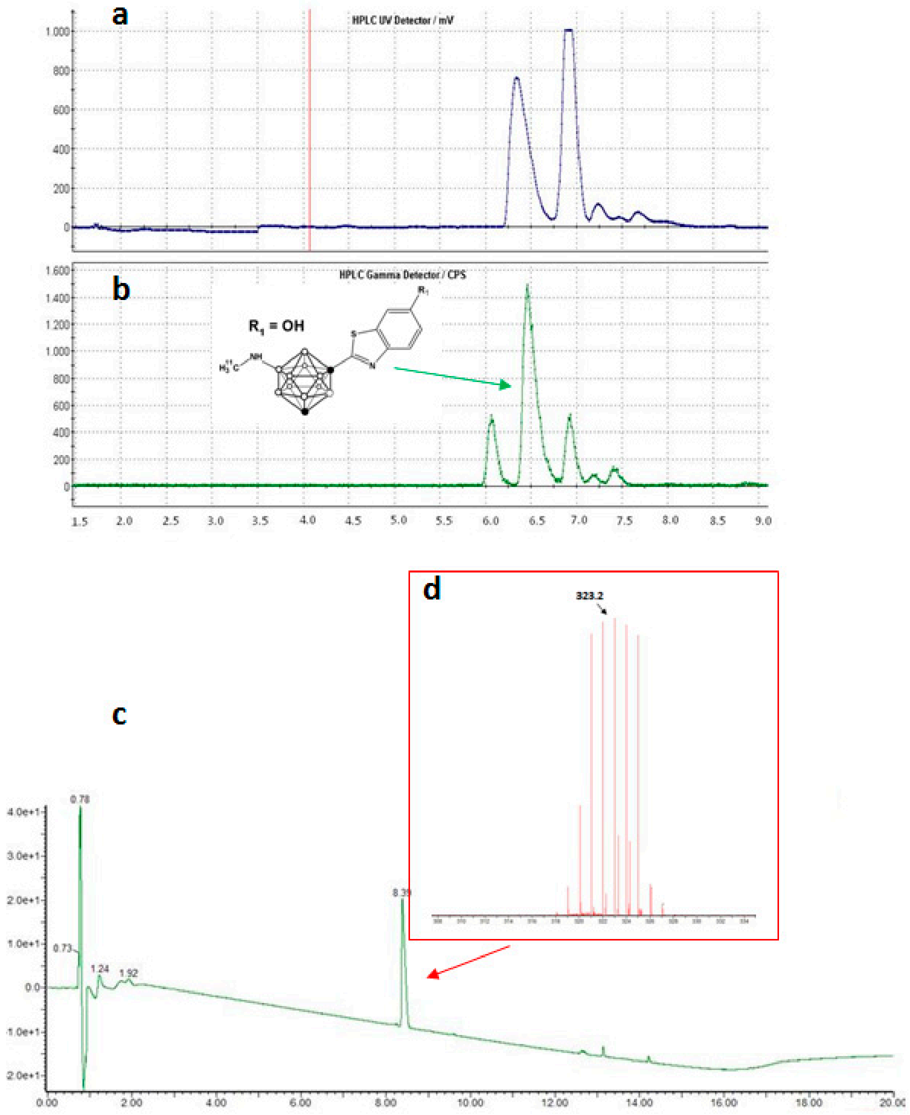

Figure S3. (**a**,**b**) Chromatographic profiles corresponding to the purification of [^{11}C]**16**: (a) UV detector and (b) radiometric detector. The collected fraction is indicated with an arrow in the radiometric profile; (**c**) chromatographic profile corresponding to LC-MS analysis of pure [^{11}C]**16** after complete decay; (**d**) MS spectrum for maximum intensity peak.

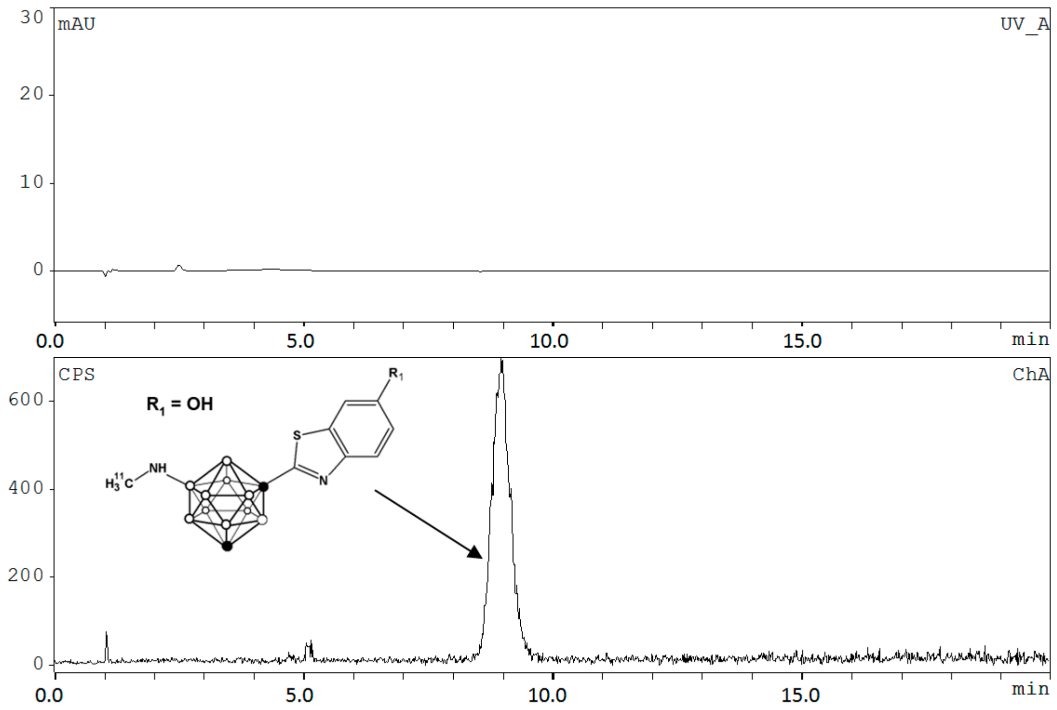

Figure S4. Chromatographic profiles corresponding to the quality control of [^{11}C]**16**: UV detector (top) and radiometric detector (bottom).
